# Differential Regulation of Cyclin E by Yorkie-Scalloped Signaling in Organ Development

**DOI:** 10.1534/g3.117.039065

**Published:** 2017-01-30

**Authors:** Zhiqiang Shu, Wu-Min Deng

**Affiliations:** Department of Biological Science, Florida State University, Tallahassee, Florida 32306-4295

**Keywords:** Yki Sd, differential regulation, cell growth and proliferation

## Abstract

Tissue integrity and homeostasis are accomplished through strict spatial and temporal regulation of cell growth and proliferation during development. Various signaling pathways have emerged as major growth regulators across metazoans; yet, how differential growth within a tissue is spatiotemporally coordinated remains largely unclear. Here, we report a role of a growth modulator Yorkie (*Yki*), the *Drosophila* homolog of Yes-associated protein (YAP), that differentially regulates its targets in *Drosophila* wing imaginal discs; whereby Yki interacts with its transcriptional partner, Scalloped (*Sd*), the homolog of the TEAD/TEF family transcription factor in mammals, to control an essential cell cycle regulator Cyclin E (CycE). Interestingly, when Yki was coexpressed with Fizzy-related (*Fzr*), a *Drosophila* endocycle inducer and homolog of Cdh1 in mammals, surrounding hinge cells displayed larger nuclear size than distal pouch cells. The observed size difference is attributable to differential regulation of CycE, a target of Yki and Sd, the latter of which can directly bind to *CycE* regulatory sequences, and is expressed only in the pouch region of the wing disc starting from the late second-instar larval stage. During earlier stages of larval development, when Sd expression was not detected in the wing disc, coexpression of Fzr and Yki did not cause size differences between cells along the proximal–distal axis of the disc. We show that ectopic CycE promoted cell proliferation and apoptosis, and inhibited transcriptional activity of Yki targets. These findings suggest that spatiotemporal expression of transcription factor Sd induces differential growth regulation by Yki during wing disc development, highlighting coordination between Yki and CycE to control growth and maintain homeostasis.

Cellular growth and proliferation are closely regulated during development and tissue homeostasis in metazoans (Neufeld *et al.* 1998; Johnston *et al.* 1999). To achieve and maintain normal organ size and morphology, cell proliferation is spatiotemporally regulated within tissues (Fowler 1986; Milán *et al.* 1996; Mao *et al.* 2013). This regulation usually involves differential expression of intrinsic factors, such as transcription factors and regulatory microRNAs, or localized extrinsic factors, such as chemical and mechanical cues (Brennecke *et al.* 2003; LeGoff *et al.* 2013; Mao *et al.* 2013). Among these factors, transcriptional regulation has been implicated as the most common way to modulate differential growth (Mannervik *et al.* 1999). During tissue development and cell differentiation, growth stimuli reiteratively use transcription factors to determine and refine cell fate, growth status, and tissue volume (Barolo and Posakony 2002). One possible scenario for differential growth regulation is that different transcription factors are harnessed by the same growth stimulus to bind to distinct DNA sequences. Therefore, spatial localizations and temporal availabilities of these transcription factors render distinct biological effects (Lelli *et al.* 2012; Slattery *et al.* 2013).

The *Drosophila* wing imaginal disc (“wing disc” hereafter) provides an ideal system to investigate how animal cells achieve differential growth and proliferation in the same tissue. This pseudostratified epithelium develops into the wing blade, wing hinge, and notum of the adult fly, during which cells in different regions of the wing disc undergo coordinated growth to achieve tissue integrity (Baena-López *et al.* 2005, Figure 1A).

**Figure 1:**
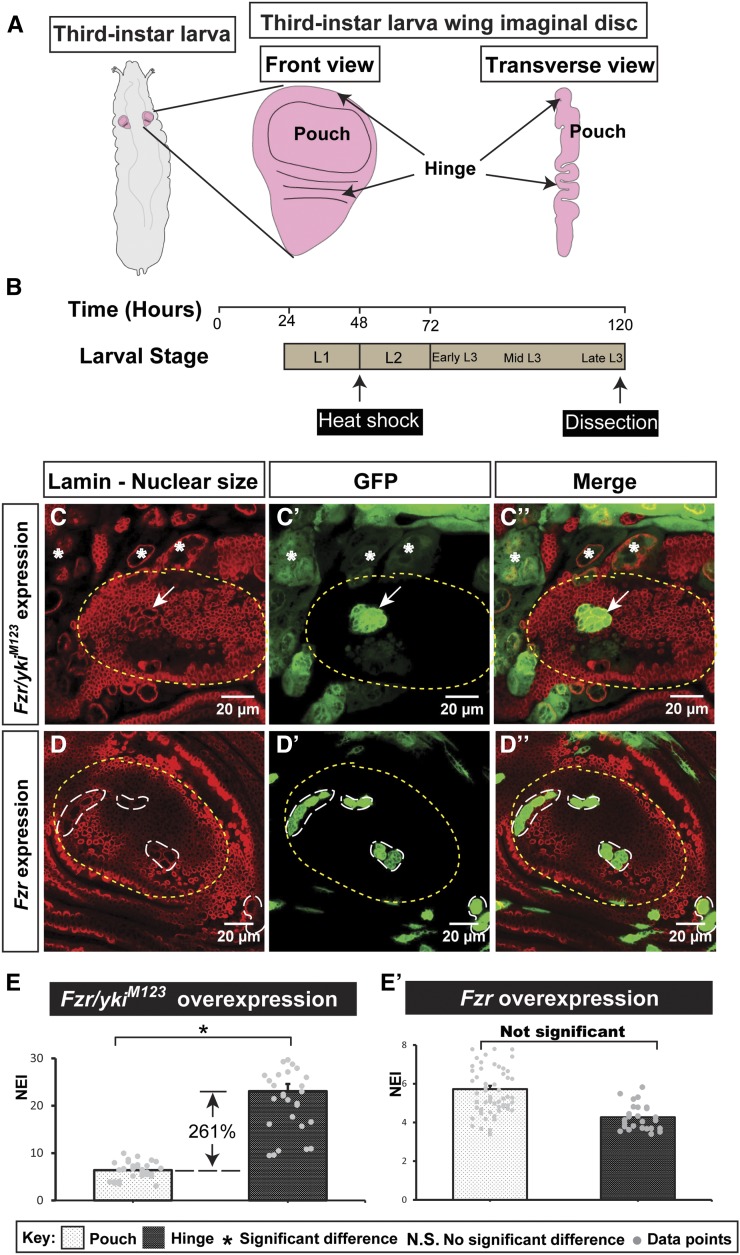
Activated Yki in endoreplicating cells shows distinct nuclear sizes in the pouch and hinge regions. (A) Diagram of *Drosophila* larva, showing the imaginal wing disc in its third-instar larval stage. (B) Larval development timeline (hours AED). Heat shock was applied at 48 hr AED and dissection was administered 120 hr AED. (C–D’’) Overexpression of Fzr and yki^M123^ (C–C’’): the difference in the nuclear size in the pouch and hinge region. Overexpression of Fzr (D–D’’) as a control. Yellow dotted lines outline the pouch. The scale bar is 20 cm. (E and E’) Quantifications of the nuclear size, demonstrating that Fzr/yki^M123^ overexpression results a larger nuclear size in the pouch than the hinge, whereas Fzr overexpression does not show significant difference [n(Fzr/yki^M123^) = 31, n(Fzr) = 62 (pouch), and 26 (hinge); error bars are SE values]. AED, after egg deposition; Fzr, Fizzy-related; GFP, green fluorescent protein; Yki, Yorkie.

Although differential growth within a tissue is generally regulated by interactions of multiple signaling pathways, a single signaling pathway can also cause distinct growth patterns in the same tissue (Van de Walle *et al.* 2013; Guo and Ohlstein 2015; Yang *et al.* 2015). To explore how differential regulation of growth can be modulated by a single signaling input, we focused on Yki, the *Drosophila* homolog of YAP and a conserved master regulator of cell growth and tissue size in metazoans (Pan 2010). Yki is the key transcription cofactor relaying information from upstream Hippo (Hpo) signaling to downstream target genes, including Death-associated inhibitor of apoptosis 1 (*Diap1*), *Expanded* (*Ex*), *CycE*, and *dMyc* (Huang *et al.* 2005; Ziosi *et al.* 2010). A dissection of the *Diap1* locus reveals a minimal sequence that mediates transcriptional regulation by Yki (Wu *et al.* 2008). Upregulation of CycE by Yki, in contrast, is less robust and less ubiquitous in the imaginal disc (Jia *et al.* 2003; Wu *et al.* 2003; Huang *et al.* 2005). As such, a mechanistic understanding of CycE regulation is lacking. Like other transcriptional coactivators, Yki does not bind to DNA directly. Two DNA-binding transcription factors, *Sd* (the homolog of the mammalian TEAD/TEF family transcription factor) and Homeothorax (*Hth*) (the homolog of the MEIS homeodomain protein), have been found to complex with Yki separately (Goulev *et al.* 2008; Wu *et al.* 2008; Zhang *et al.* 2008; Peng *et al.* 2009). Sd physically interacts with Yki and is required for cell proliferation induced by Yki (Goulev *et al.* 2008; Zhang *et al.* 2008). Hth, on the other hand, has been shown to be a transcription factor of Yki in the eye disc that regulates microRNA *bantam* to promote proliferation and protect eye progenitor cells from apoptosis (Peng *et al.* 2009). Recently, evidence for differential regulation by Yki that involves Sd and Hth in the wing and eye discs, respectively, has been described (Slattery *et al.* 2013), suggesting that the availability of transcription factors contributes to tissue-specific signaling outputs. However, it remains to be determined whether Yki discriminates between these two transcription factors in the same tissue in regulating cell growth and proliferation.

Tissue growth derives primarily from an increase in cell number through cell proliferation. One avenue for Yki to affect the cell cycle is through regulating CycE, which promotes G1-to-S transition and can induce extra rounds of mitosis in *Drosophila* (Knoblich *et al.* 1994; Huang *et al.* 2005; Bloom and Cross 2007). Tissue growth can also originate from endoreplication, a common cell cycle alternative in which cells replicate their genomic DNA without cell division (Edgar and Orr-Weaver 2001; Klusza and Deng 2011; Tamori and Deng 2014). Misexpression of *Fzr*, the *Drosophila* homolog of *Cdh1* in mammals, can force the cell cycle from mitosis into endocycle, the mechanism of which occurs as Fzr activates the Anaphase Promoting Complex/Cyclosome (APC/C), and the Fzr/APC complex degrades mitotic cyclins, such as cyclins A, B, and B3, via the ubiquitin-mediated proteolytic pathway (Sigrist and Lehner 1997; Schaeffer *et al.* 2004; Narbonne-Reveau *et al.* 2008). Recently, Yki has been shown to regulate polyploidization and cell fusion to heal wounds in the *Drosophila* epidermis, and compensate for cell loss in mouse corneal endothelium through upregulating its targets (Losick *et al.* 2013, 2016).

Here, we report that Yki spatiotemporally regulates CycE through Sd. The pouch cells and hinge cells exhibited dramatic differences in cell and nuclear sizes when endoreplication was induced in Yki-activated cells. The regulation of differential growth by Yki depends on the temporal and spatial expression of Sd during wing disc development. Excessive CycE negatively feeds back to Hpo signaling by limiting transcription activity of Yki targets *Diap1* and *CycE*. Our studies reveal how Yki signaling controls tissue/organ growth through differential regulation of cell cycle genes.

## Materials and Methods

### Fly stocks and genetics

All flies were maintained at 25°. To generate transgene overexpression in disc cells, the flip-out Gal4 (*hsFLP*; *actin > CD2 > Gal4*) was crossed to flies that carry an RNAi or an overexpression construct under the UAS promoter. A construct of UAS-GFP, which serves as a marker for the overexpression, was always included in the cross. Larval progeny were heat shocked at 37° for 15 min, 2 d after egg deposition (AED). The progeny flies were kept at 25° for 3 d before dissection.

The temporal and regional gene expression targeting (TARGET) technique is described in McGuire *et al.* (2004), in which Gal80 is temperature-sensitive. The parental flies were cultured at 18°, during which Gal80 actively suppresses transgene expression, until the progeny flies develop into second-instar larvae. Then these larvae were transferred to 29° to inactive Gal80. At this stage, transgene genes including GFP were expressed.

The MARCM technique is described in Lee and Luo (2001), by which each fluorescent group of cells is generated via proliferating from a single progenitor cells. Therefore, the number of cells in a clone is positively correlated with the proliferation rate of the cells during a given timeframe. Flies were treated according to Figure 1B.

The following fly stocks were used in this study: *UAS-Fzr* (a gift from C. Lehner); *UAS-yki^M123^*, *UAS-Sd (active form)*, *UAS-Sd-RNAi*, and *Diap1-GFP-4.3(3)* (gifts from J. Jiang); *UAS-Hth (full length)* (a gift from R. Mann); *Hth:YFP* (CPTI-001356, The FlAnnotator, a gift from F. Casares); and *UAS-dMyc* (a gift from L. Johnston). *UAS-CycE*, *UAS-Sd*, *UAS-Hth-RNAi*, *UAS-Diap1*, *Ex-lacZ*, *CycE-lacZ*, *UAS-Dacapo*, and *tubP-Gal80^ts^* were obtained from the Bloomington *Drosophila* Stock Center.

### Immunocytochemistry

Wing imaginal discs were dissected in 1 × phosphate-buffered saline (PBS) and fixed by shaking for 15 min in 4% paraformaldehyde at room temperature. They were rinsed with PBT three times for 15 min each, and blocked in PBTG (goat serum) for 1 hr at room temperature. The discs were then incubated with primary antibodies overnight at 4°. The next day, discs were rinsed with PBT three times for 15 min each. They were then incubated with a secondary antibody for 2 hr at room temperature. They were rinsed with PBT for 15 min before staining with DAPI (1:1000, Invitrogen) for 15 min at room temperature. The discs were rinsed with PBS three times for 15 min each and dissected onto slides in 70% glycerol.

The following antibodies were used: rabbit anti-β-galactosidase (1:2000, MP Biomedicals), mouse anti-BrdU (1:30, BD Biosciences), rabbit anti-phospho-Histone H3 (1:200, Millipore), mouse anti-lamin (1:200, the Development Studies Hybridoma Bank), mouse anti-Eya (1:10, the Development Studies Hybridoma Bank), rabbit anti-Dcp1 (1:200, Cell signaling), and guinea pig anti-Sd (1:500, a gift from K. Guss). Secondary antibodies were Alexa 488, 546, or 633 goat anti-mouse (1:500) and Alexa 488, 546, or 633 goat anti-rabbit (1:500) (Molecular Probes). Images were captured on a Zeiss LSM-800 confocal microscope. Figures were processed and arranged in Image J and Adobe Illustrator.

### Time course of wing disc dissection

To synchronize development of progenies, 50 female and 10 male wild-type flies were collected and cultured in 25° for 4 hr. Then, parental flies were transferred out and the development time was set as 2 hr AED. We dissected larvae at different hours AED points and acquired wing imaginal disc confocal images. Using Image J, we measured the area of the wing disc at different time points, and at least five wing discs were considered. We used log2 to transform the data and plotted the growth curve of the wing imaginal disc. Our results are comparable to a previous study (Nienhaus *et al.* 2012).

### Quantitative analysis

#### Nuclear Enlargement Index (NEI) calculation:

We drew closed circles along the lamin staining, which outlines the nuclei, and measured the area of each circle as its nuclear size. For each wing disc, the average nuclear size of 10 random wild-type cells was used as the control. NEI of a cell is the ratio of its nuclear size to the wild-type control. Ten random circles were drawn in the transgene-expressing cells in each disc and at least five wing discs were counted for each genotype. We avoided obscure regions. The average ratio was calculated as the NEI of a genotype.

#### Reporter signal intensity measurement:

In each disc, we drew 10 random, equally sized circles in both the transgene-expressing region and the wild-type region, and measured the signal intensity of each circle. The average intensity of 10 random circles in the wild-type region was considered as the control, and the relative intensity of a transgene-expressing circle was the ratio of its intensity to this control. The reporter signal intensity of a genotype was calculated as the average of all the ratios across at least five discs of this genotype. We avoided obscure regions.

#### The BrdU incorporation and phospho-Histone H3 (pH 3) staining analyses:

BrdU incorporation assay was conducted as described in Sun and Deng 2005. In each disc, we counted the BrdU incorporation and pH 3 staining events, and estimated the number of cells. We solely focused on the pouch region. To estimate the number of cells, we drew a random circle within the region of interest and measured its area. Then we counted the number of cells within this circle and calculated the ratio of the cell number to the area. Using this ratio, we were able to calculate the approximate number of cells in the entire region. At least five discs were counted.

#### Statistical analysis:

Two-tailed unpaired *t*-tests assuming unequal variances were performed for all statistical analyses. *P* < 0.05 was considered statistically significant for all analyses.

### Data availability

Fly strains and reagents are available upon request. The authors state that all data necessary for confirming the conclusions presented in the article are represented fully within the article.

## Results

### Activated Yki in endoreplicating cells shows distinct nuclear sizes in the pouch and hinge regions

Yki induces tissue overgrowth through activating proproliferative factors like dMyc and CycE, and antiapoptotic factors like Diap1 (Huang *et al.* 2005; Ziosi *et al.* 2010). To assess gene functions, we administered the heat shock 48 hr AED to induce ectopic gene expression and dissected the wing disc 120 hr AED (Figure 1B). Ectopic expression of *yki^M123^*, an active form of Yki that bears Serine to Alanine (S/A) mutations at S168, S169, and S171 sites (Zhang *et al.* 2008), generates the overgrowth phenotype and artificial folds in the wing disc (Supplemental Material, Figure S1 in File S1; see File S2 for corresponding legends to supplemental figures and table, using the MARCM technique, more details in *Materials and Methods*). In an effort to compare the difference between cells in distinct regions of the wing disc on Yki activation, we expressed Fzr, which promotes degradation of mitotic cyclins and thus induces endoreplication (Schaeffer *et al.* 2004), together with *yki^M123^*. We found that the hinge cells had much larger nuclei than the pouch cells (Figure 1, C–C’’). As a control, misexpression of Fzr alone did not show significant difference in nuclear sizes between the pouch and hinge areas (Figure 1, D–D’’). To quantify the effect on nuclear size by different genetic combinations, we introduced the NEI to indicate the average multiple of the nuclear sizes of the genetically-manipulated cells to that of neighboring wild-type cells. We found that *Fzr/yki^M123^*-coexpressing pouch cells (NEI = 6.39) were significantly smaller than the hinge cells (NEI = 23.07, Figure 1E). In contrast, the NEI of the cells with Fzr misexpression alone was not statistically different between the pouch and hinge regions (Figure 1E’). Taken together, these findings suggest that Yki signaling may affect the cell cycle differently between the pouch and hinge regions during wing disc development.

### Yki regulates its targets differentially in the wing disc

**Figure 2:**
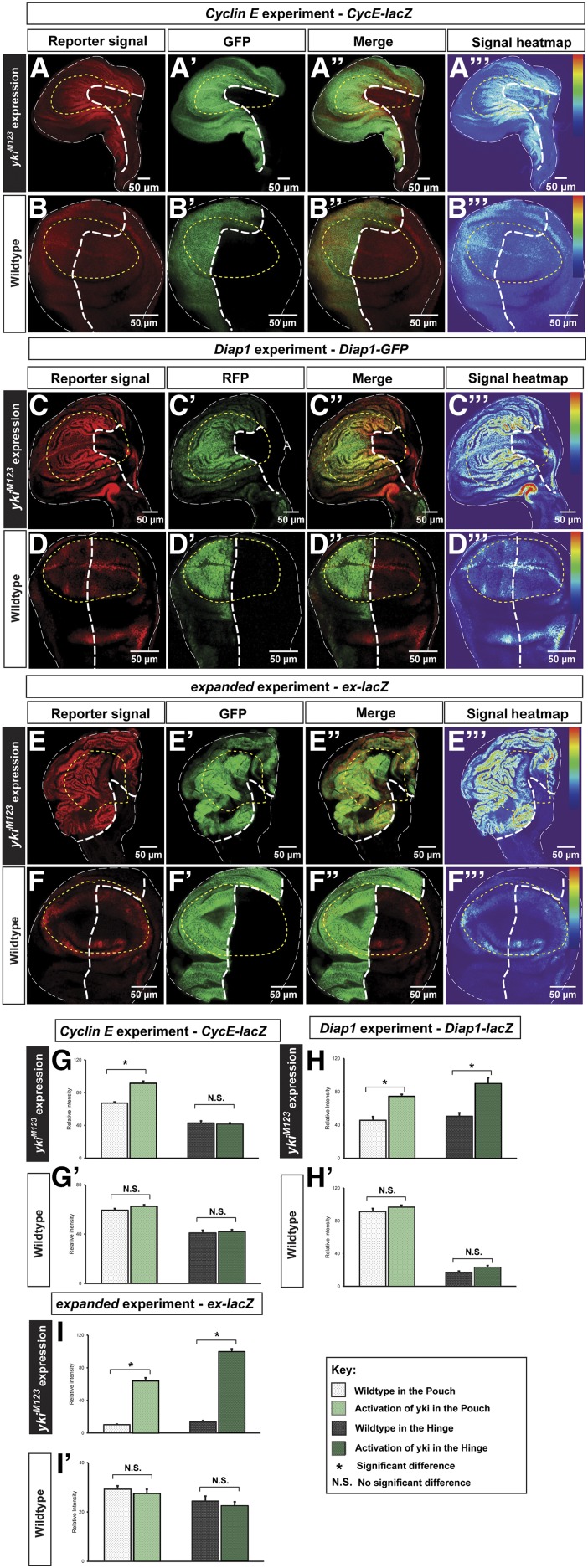
Ectopically-expressed Yki differentially upregulates CycE in the wing disc. (A–B’’’) The CycE reporter *CycE-lacZ* experiments: (A) *CycE-lacZ* was examined upon ectopic activation of Yki (A’). *CycE-lacZ* shows a higher signal intensity in the pouch region (A and A’’’). As a control, in the wild-type wing disc, the CycE reporter shows the endogenous pattern of CycE (B and B’’’). (C–D’’’) The Diap1 reporter *Diap1-GFP* experiments: (C) was examined upon ectopic activation of Yki (C’). *Diap1-GFP* shows signals with higher intensity in the *yki^M123^* expressing cells than the wild-type cells and this upregulation is in both pouch and hinge (C and C’’’). As a control, in the wild-type wing disc, the Diap1 reporter shows the endogenous pattern of Diap1 (D and D’’’). (E–F’’) the expanded reporter *ex-lacZ* experiments: (E) *ex-lacZ* was examined upon ectopic activation of Yki (E’). *ex-lacZ* shows signals with higher intensity in the *yki^M123^* expressing cells than the wild-type cells and this upregulation is in both pouch and hinge (E and E’’’). As a control, in the wild-type wing disc, the expanded reporter shows the endogenous pattern of ex (F and F’’’). The scale bar is 50 cm. (G–I’) Quantifications of the signal intensity of *CycE-lacZ*, *Diap1-GFP*, and *ex-lacZ* demonstrating that Yki activation fails to significantly upregulate CycE in the hinge (*n* = 10, error bars are SE values). CycE, Cyclin E; GFP, green fluorescent protein; N.S., no significant difference; RFP; red fluorescent protein; Yki, Yorkie.

To examine whether different regions in the wing disc respond differently to Yki activation, we assessed the expression of Yki target genes using reporters, *i.e.*, *ex-lacZ*, *Diap1-GFP*, and *CycE-lacZ*. Using tissue-specific Gal4 lines, *engrailed* (*en*)-*Gal4* and *Hedgehog* (*Hh*)-*Gal4*, to drive *yki^M123^* misexpression, we found that the Yki-activating compartment had dramatic overgrowth and Gal4 lines were larger in tissue size than their wild-type counterparts (Figure 2, A, C, E, and G), supporting the link between Yki activation and tissue overgrowth (Huang *et al.* 2005; Dong *et al.* 2007). While all three targets we examined were upregulated in the *yki^M123^*-misexpressing compartment, the effects of *CycE* elevation were more pronounced in the pouch region compared to the rest of the disc (Figure 2A, relative signal intensity is shown as the heat map in Figure 2A’’’, see *Materials and Methods* for details). We also note that upregulation of CycE by Yki is not entirely restricted in the pouch area. In contrast, upregulations of *Diap1* and *ex* seemed indiscriminate between the pouch and hinge regions (Figure 2, C and D). To quantify the results, we took the signal intensity measurements (described in *Materials and Methods*) and verified that, while *Diap1* and *ex* were significantly upregulated by Yki activation in both the pouch and hinge regions (Figure 2, H–H’ and I–I’), *CycE* was less responsive to Yki activation in the hinge than in the pouch region, and upregulation of *CycE* in the hinge upon Yki activation was not significant (Figure 2, G–G’). Taken together, our findings suggest that Yki regulates its target *CycE* differentially between the pouch and hinge regions, which may be responsible for the size differences of *Fzr/yki^M123^* cells between these two regions.

### CycE suppresses Fzr-induced endoreplication

**Figure 3:**
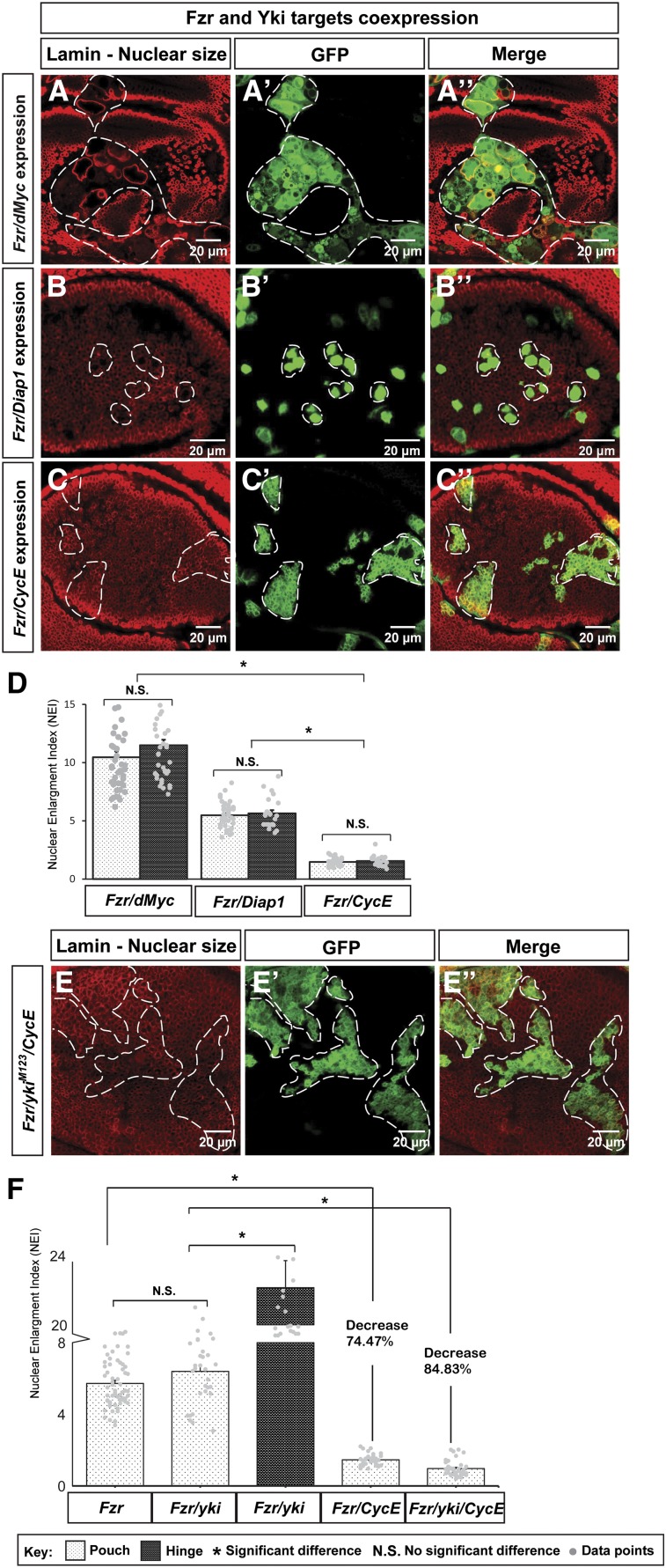
CycE sufficiently suppresses Fzr-induced endoreplication. (A–C’’) Overexpression of *Fzr/dMyc* (A–A’’), *Fzr/Diap1* (B–B’’), and *Fzr/CycE* (C–C’’) were examined in the wing discs. The scale bar is 20 cm. (D) Quantifications of NEIs of *Fzr/dMyc*, *Fzr/Diap1*, and *Fzr/CycE*. In each genetic combination, the comparisons between the cells in the pouch and in the hinge show no significant difference. However, NEI of *Fzr/CycE* is significant lower than that of *Fzr/dMyc* and *Fzr/ Diap1* [n(*Fzr/dMyc*) = 44 (pouch), 40 (hinge); n(*Fzr/Diap1*) = 36 (pouch), 22 (hinge); and n(*Fzr/CycE*) = 37 (pouch), 21 (hinge); error bars are SE values, gray dots are data points]. (E–E’’) Overexpression of *Fzr/yki^M123^/CycE* was examined in the wing disc. The scale bar is 20 cm. (F) Quantifications of NEIs of pouch Fzr-expressing cells, pouch *Fzr/yki^M123^*-expressing cells, hinge *Fzr/yki^M123^*-expressing cells, pouch Fzr/CycE-expressing cells, and pouch *Fzr/yki^M123^/CycE*-expressing cells [n(*Fzr*) = 62, n(*Fzr/yki^M123^*) = 31, n(*Fzr/CycE*) = 37, and n(*Fzr/yki^M123^/CycE*) = 38; error bars are SE values, gray dots are data points]. CycE, Cyclin E; Fzr, Fizzy-related; GFP, green fluorescent protein; NEI, nuclear enlargement index; N.S., no significant difference; Yki, Yorkie.

Since *Fzr/yki^M123^*-coexpressing cells showed distinct nuclear sizes in different regions of the wing disc (Figure 1, C–C’’ and E), we asked whether coexpression of *Fzr* with a Yki target gene, *e.g.*, *Diap1*, *dMyc*, or *CycE*, would also result in nuclear size differences between the pouch and hinge regions. These Yki targets play critical roles in cell survival and proliferation (Knoblich *et al.* 1994; Johnston *et al.* 1999; Wang *et al.* 1999), and their ectopic expression alone did not change the nuclear size (Figure S2 in File S1). When coexpressed with *Fzr*, no significant differences in nuclear sizes were observed between the pouch and hinge regions in these three genetic combinations (*Fzr/Diap1*, *Fzr/dMyc*, and *Fzr/CycE*; Figure 3, A–C, quantified in Figure 3D). However, *Fzr/CycE*-coexpressing cells had much smaller nuclei than the other two coexpressions, mimicking the small nuclear size of *Fzr/yki ^M123^*-expressing cells in the pouch region of the wing disc (Figure 3D). Further statistical analyses showed that while *Fzr/yki^M123^* coexpression had a similar NEI to that of *Fzr* expression alone in the pouch, addition of CycE in *Fzr*-misexpressing cells significantly lowered the NEI in the pouch (NEI = 1.46, Figure 3F), suggesting that upregulated CycE can suppress Fzr-induced endoreplication. In contrast, *Fzr/dMyc* had an increase in the NEI upon *dMyc* overexpression (NEI = 10.46, Figure 3, A and D) comparing to that of Fzr overexpression alone (NEI = 5.73), consistent with the role of dMyc in promoting cell mass in both mitosis and endoreplication (Johnston *et al.* 1999; Pierce *et al.* 2004). Another target, Diap1, whose role is to inhibit caspase activity to maintain cell survival (Wang *et al.* 1999), had no significant effect on the NEI (NEI = 5.48) when coexpressed with *Fzr* (NEI = 5.73, Figure 3, B and D). To determine whether CycE can regulate the nuclear size difference of the *Fzr/yki^M123^*-expressing cells, we misexpressed *Fzr*, *yki^M123^*, and *CycE* simultaneously and found that the NEI was also significantly reduced compared to that of *Fzr/yki^M123^* coexpression in the pouch (Figure 3F), but similar to that of *Fzr/CycE* coexpression (Figure 3E). Combining the previous results on different spatial patterns of target genes upon Yki misexpression (Figure 2, A, C, and E), these results suggest that CycE acts downstream of Yki in limiting Fzr-induced endoreplication in the pouch region of the wing disc.

### Yki harnesses Sd to regulate CycE, and inhibits endoreplication

**Figure 4:**
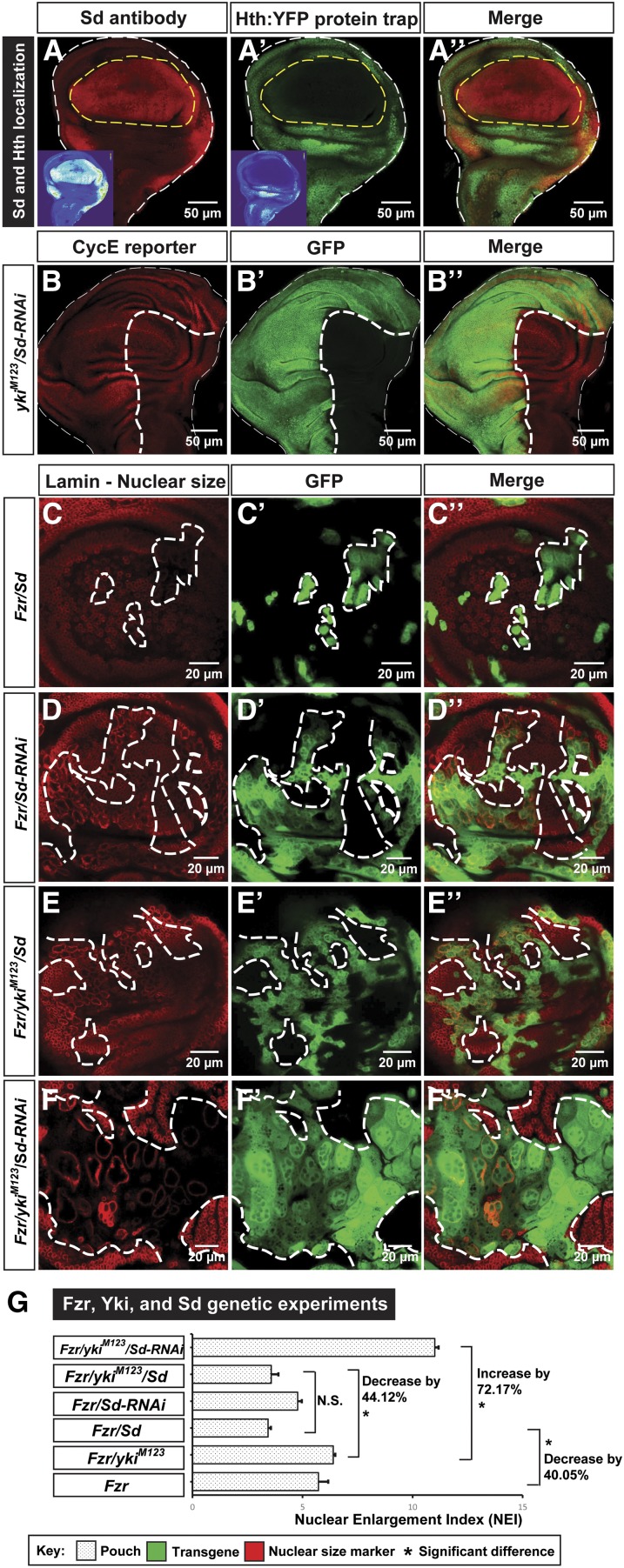
Yki uses Sd to regulate CycE, and inhibits endoreplication. (A–A’’) Sd and Hth expression patterns in the wing disc were examined using an Sd antibody [(A), shown in red and a Hth:YFP protein trap strain (A’), shown in green]. Signal heat maps are inset accordingly. The scale bar is 50 cm. (B–B’’) Effect of knocking down Sd in Yki-overexpressing cells was examined using a CycE reporter *CycE-lacZ*. The scale bar is 50 cm. (C–F’’) The effects of Sd on the nuclear size were examined through genetic experiments, including *Fzr/Sd*, *Fzr/Sd-RNAi*, *Fzr/yki^M123^/Sd*, and *Fzr/yki^M123^/Sd-RNAi*. The scale bar is 20 cm. (G) Quantifications of NEIs of Sd genetic studies in the wing pouch. Significant differences were observed between *Fzr/yki^M123^* and *Fzr/yki^M123^/Sd*, and *Fzr/yki^M123^* and *Fzr/yki^M123^/Sd-RNAi* [n(*Fzr*) = 62, n(*Fzr/yki^M123^*) = 31, n(*Fzr/Sd*) = 36, n(*Fzr/Sd-RNAi*) = 93, n(*Fzr/yki^M123^/Sd*) = 96, and n(*Fzr/yki^M123^/Sd-RNAi*) = 68; error bars are SE values]. CycE, Cyclin E; Fzr, Fizzy-related; GFP, green fluorescent protein; Hth, Homeothorax; NEI, nuclear enlargement index; RNAi, RNA interference; Sd, Scalloped; Yki, Yorkie.

CycE thus emerges as a critical target for Yki to differentially regulate cell growth. Next, we examined how Yki may regulate CycE differentially in the wing disc. Previous studies have revealed the role of Sd and Hth as transcription factors of Yki to control its target gene expression (Wu *et al.* 2008; Zhang *et al.* 2008). Interestingly, by using an Sd antibody (Guss *et al.* 2013) and a Hth protein trap line (from Flannotator, CPTI-001356), we found that in third-instar larval discs, Sd was primarily expressed in the wing pouch and Hth was expressed in the wing hinge and notum regions (Figure 4, A–A’’). Based on their distinct expression patterns in the wing disc and a recent finding that Sd and Yki induce compensatory proliferation after damage by upregulating CycE (Meserve and Duronio 2015), we hypothesized that Yki-regulated CycE expression is Sd-dependent. Indeed, a genome-wide ChIP-seq analysis showed that Sd, but not Hth, regulates the mitotic cell cycle (Slattery *et al.* 2013). Using the ChIP-seq database (Slattery *et al.* 2013), we reanalyzed the *CycE* sequence and the 16.4 kb of the 5′ *CycE* regulatory sequence, due to their effect on *CycE* transcription regulation (Jones *et al.* 2000), and found seven potential Sd-binding peaks (Table S1 in File S1), suggesting that Sd is capable of binding the *CycE* sequences and thus affect the cell cycle.

To determine whether Sd is involved in Yki-dependent CycE regulation, we knocked down Sd via Sd-RNAi lines and found that the upregulation of the *CycE-lacZ* reporter by Yki activation was largely reduced (Figure 4, B–B’’). Knockdown of Sd (Sd-RNAi) by itself also showed a mild downregulation of *CycE* in the wing disc (Figure S3, A–A’’’ in File S1). To test whether Sd affects Fzr-induced endoreplication by regulating CycE, we overexpressed Sd in Fzr-expressing cells, and found that the NEI (= 3.43) was decreased by 40.05% when compared with that of Fzr misexpression alone (Figure 4C, quantified in Figure 4G), probably through the upregulation of CycE by Sd. As controls, neither Sd overexpression nor Sd-RNAi by itself affected the nuclear size (Figure S3, B–B’’ and C–C’’ in File S1). Taken together, our findings indicate that Sd overexpression impedes Fzr-induced endoreplication.

If Sd is required for Yki to regulate CycE, the NEI of *Fzr/yki^M123^*-expressing cells should be dependent on the Sd level. Indeed, we found that addition of Sd significantly decreased (NEI = 3.57) the NEI of *Fzr/yki^M123^*-expressing cells, and that removal of Sd significantly increased (NEI = 11.01) the NEI of *Fzr/yki^M123^*-expressing cells (Figure 4, E and F, quantified in Figure 4G). Moreover, the NEI of *Fzr/yki^M123^/Sd*-overexpressing cells is close to that of Fzr/Sd-expressing cells in the pouch, suggesting that Sd is capable of limiting the proliferative effect induced by Yki (Figure 4G). To explore the possibility that Hth may affect the NEI of *Fzr/yki^M123^*-expressing cells, we expressed Hth in the *Fzr/yki^M123^*-expressing cells and found that overexpression of *Fzr/yki^M123^/Hth* led to an increase of NEI (= 8.08) when compared with that of *Fzr/yki^M123^*, suggesting that Hth does not contribute to the small nucleus phenotype of *Fzr/yki^M123^* cells in the pouch (Figure S4, A–A’’ in File S1, quantified in Figure S4D in File S1). Of note, Hth overexpression generated ectopic folds in the imaginal disc (Figure S4, B–B’’ in File S1), supporting the role of Hth in hinge specification (Casares and Mann 2000), whereas depleting Hth was not sufficient to transform hinge cells into pouch cells (Figure S4, C–C’’ in File S1). Together, our results indicate that Sd is key to Yki in regulating CycE in the pouch, and that this regulation alleviates Fzr-induced endoreplication.

### Temporal regulation of CycE by Yki

**Figure 5:**
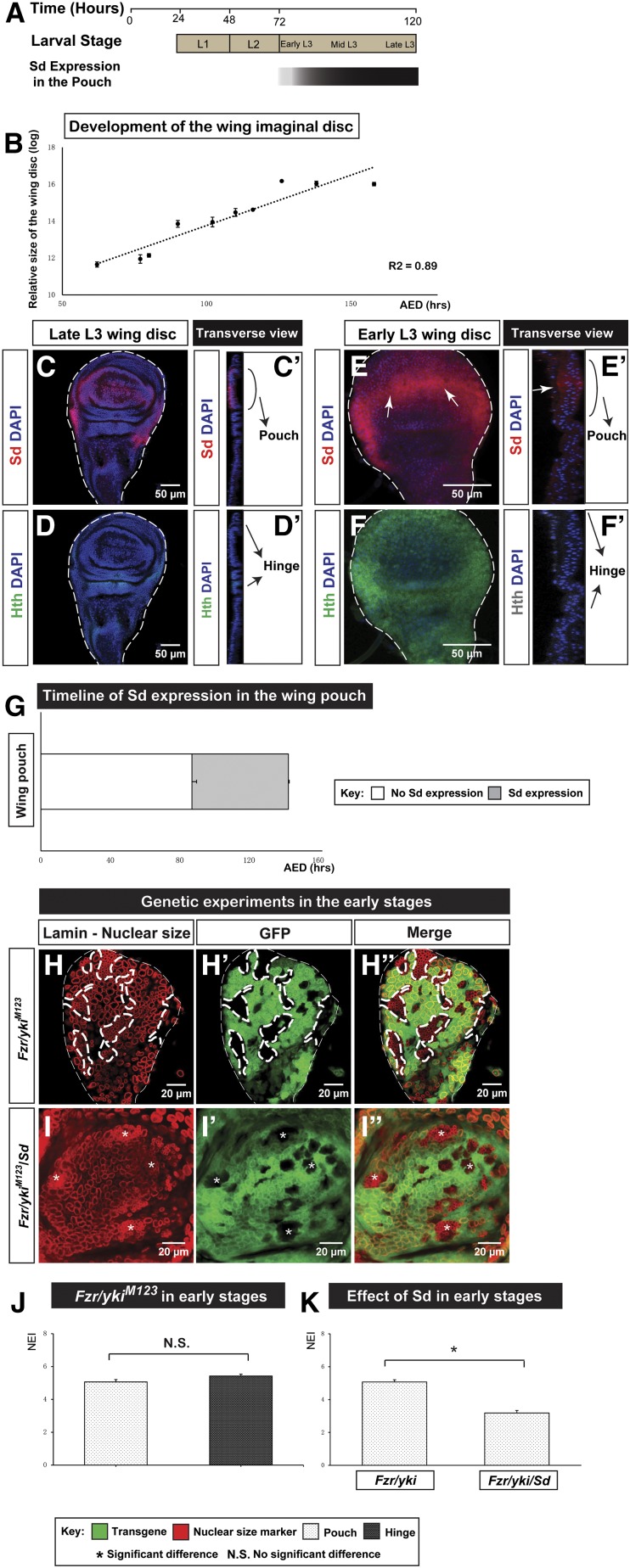
Yki regulates CycE during development through temporally expressed Sd. (A) Timeline of Sd expression during larval development, noting that Sd is predominantly expressed in the wing pouch region. (B) A wing disc growth curve depicts the relationship between the overall size of the wing (in log) and time (hours AED) of development. A regression equation (shown in a dotted line), *y* = 0.0547*x* + 8.2974, is built with an *R*-square = 0.89. Error bars are SE values. (C–F’) Expression patterns of Sd and Hth in the wing disc of the late and early third-instar larval stage (L3). Sd is expressed predominantly in the wing pouch in late L3 (C–C’) and begins to emerge in early L3 [(E and E’), arrows show the emergence of Sd]. Hth is primarily expressed in the hinge in late L3 (D and D’) and ubiquitously expressed in early L3 (F and F’). The transverse view shows the structure of the wing disc, helping determine the localization of Sd and Hth. The scale bar is 50 cm. (G) Timeline of Sd expression in the wing pouch is established by the wing disc growth curve and Sd staining. Error bars are SE values. (H–I’’) The temporal phenotypes of *Fzr/yki^M123^* and *Fzr/yki^M123^/Sd* cells were examined in the early stage of larval development. The scale bar is 20 cm. (J) Quantifications of NEIs of *Fzr/Yki^M123^* overexpression in the pouch and hinge in the early stage (n = 72, error bars are SE values). (K) Quantifications of NEIs of *Fzr/yki^M123^* and *Fzr/yki^M123^/Sd* overexpression in the early stage [n(*Fzr/yki^M123^*) = 72 and n(*Fzr/yki^M123^*/*Sd*) = 20; error bars are SE values]. AED, after egg deposition; CycE, Cyclin E; DAPI, 4’,6-diamidino-2-phenylindole; Fzr, Fizzy-related; GFP, green fluorescent protein; Hth, Homeothorax; NEI, nuclear enlargement index; Sd, Scalloped; Yki, Yorkie.

Hth is uniformly expressed in the wing disc during the first- and second-instar larval stages, until it is inhibited by Sd and Vestigial (*Vg*) for pouch formation (Azpiazu and Morata 2000; Casares and Mann 2000; Figure 5A). Given that Sd is only expressed after the late second-instar stage, we asked whether *Fzr/yki^M123^* coexpression can induce different nuclear sizes prior to Sd expression in the presumptive wing pouch region. To assess the developmental stage of wing discs, we established a growth model for wing disc development by recording its size at different developmental stages (hours AED; Figure S5 in File S1). The regression curve between these two variables (Figure 5B) is consistent with the results from an *in vivo* imaging study of wing disc development (Nienhaus *et al.* 2012). With this growth model as a benchmark, we confirmed that Hth is ubiquitously expressed in the entire wing disc until Sd emerges in the pouch approximately at 85.90 hr AED (Figure 5, C–F, quantified in Figure 5G). We examined the nuclear size of *Fzr/yki^M123^*-coexpressing cells at the pre-Sd stage (83.63 hr AED), and found no significant difference in NEI between the presumptive pouch (NEI = 5.07) and hinge (NEI = 5.43, Figure 5H, quantified in Figure 5J) regions, suggesting that Sd expression is indispensable for the nuclear size differences between the pouch and hinge. To further corroborate this, we overexpressed Sd together with *Fzr* and *yki^M123^* in wing disc cells during early larval development, and found that the NEI (=3.18) of *Fzr/yki^M123^/Sd*-expressing cells was significantly lower than that of *Fzr/yki^M123^* (Figure 5I, quantified in Figure 5K). Taken together, our findings suggest that temporally expressed Sd during disc development contributes to the temporal regulation of CycE.

### CycE promotes cell proliferation and cell death

**Figure 6:**
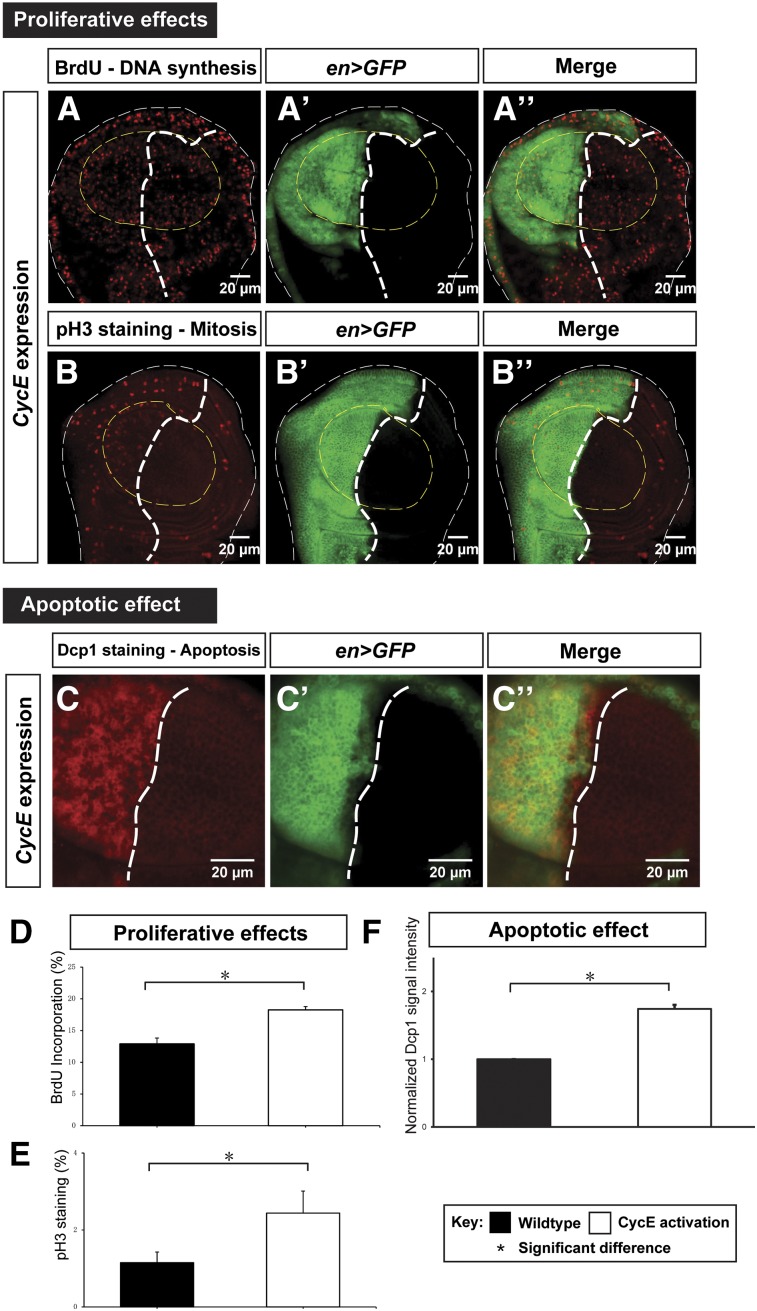
Ectopic CycE promotes both proliferation and apoptosis. (A–B’’) DNA synthesis activity and mitotic activity upon CycE ectopic expression were examined through the BrdU incorporation assay (A–A’’) and pH 3 staining (B–B’’), respectively. The scale bar is 20 cm. (C–C’’) The apoptotic effect upon CycE overexpression in the wing disc was examined by Dcp1 staining. The scale bar is 20 cm. (D–F) Quantifications of the proliferative and apoptotic effects upon CycE overexpression. Percentages of cells with BrdU incorporation events (D) and pH 3 staining signal (E), and the apoptotic effect by normalized Dcp1 signal intensity (F) are shown [n(BrdU) = 8; n(pH 3) = 6; and n(Dcp1) = 35; error bars are SE values]. BrdU, bromodeoxyuridine; CycE, Cyclin E; GFP, green fluorescent protein; pH 3, phospho-Histone H3.

The results presented so far indicate that CycE is spatiotemporally regulated by Yki through a transcriptional mechanism. To determine whether differential regulation of CycE contributes to differential cell proliferation in the wing disc, we overexpressed CycE in different disc regions using tissue-specific Gal4s and the TARGET technique, which allows temporal gene expression through manipulated environment temperature (McGuire *et al.* 2004). Overexpression of CycE did not produce tissue overgrowth in the third-instar larval disc as observed in Yki activation, suggesting that CycE is insufficient to replicate the Yki phenotype (Figure 6, A and B). To quantitatively measure and compare the proliferation rate in different genetic backgrounds, we measured the BrdU incorporation activity, which reports DNA synthesis, and the signal of the mitotic marker pH 3 in wing pouches with altered CycE levels. Overexpression of CycE significantly increased percentages of cells with BrdU and pH 3 staining (Figure 6, A and B, quantified in Figure 6, D and E), indicating its ability to promote DNA synthesis and accelerate the cell cycle, consistent with the critical role of CycE in promoting S phase entry (Knoblich *et al.* 1994). Employing the MARCM technique (Lee and Luo 2001, more details in *Materials and Methods*), we further confirmed that CycE-overexpressing clones had significantly more cells per clone than the wild-type control (Figure S6 in File S1). In addition, we found that CycE overexpression resulted in more apoptosis (Figure 6C, quantified in Figure 6F). Together, our results suggest that CycE facilitates cell proliferation by promoting DNA synthesis and cell division, linking differential regulation of CycE by Yki with differential proliferation rates.

### CycE negatively feeds back to Yki targets

**Figure 7:**
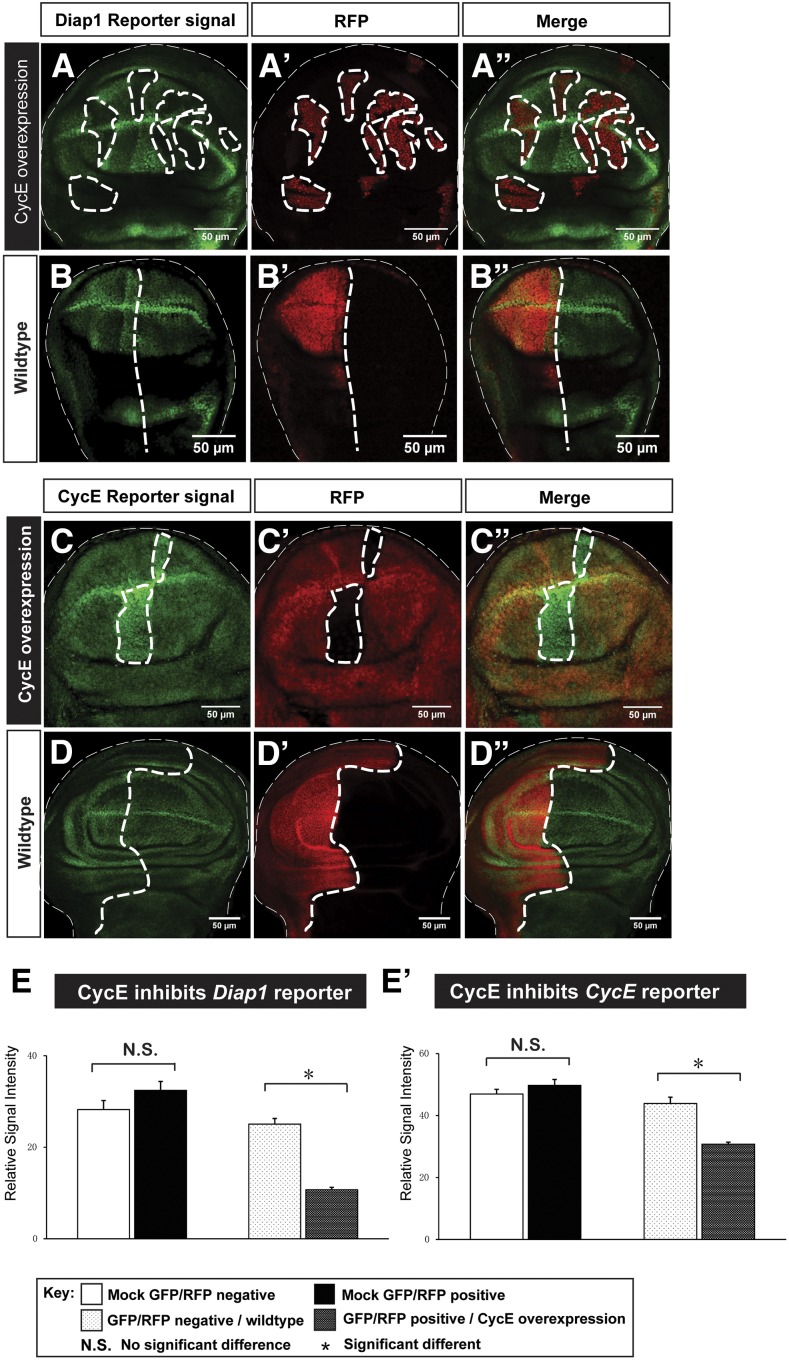
CycE inhibits transcriptional activity of Yki targets. (A–B’’) the Diap1 reporter *Diap1-GFP* was downregulated upon CycE overexpression (A–A’’). As a control, expression of mock RFP shows no effects on *Diap1-GFP* (B–B’’). (C–D’’) the CycE reporter *CycE-lacZ* was downregulated upon CycE overexpression (C–C’’). As a control, expression of mock GFP shows no effects on *CycE-lacZ* (D–D’’). The scale bar is 50 cm. (E and E’) Quantifications of the reporter activity upon CycE overexpression. *Diap1-GFP* (E) and *CycE-lacZ* (E’) signal intensity were significantly reduced when CycE is overexpressed [n(*Diap1-GFP*) = 20 and n(*CycE-lacZ*) = 25; error bars are SE values). CycE, Cyclin E; GFP, green fluorescent protein; N.S., no significant difference; RFP, red fluorescent protein; Yki, Yorkie.

Feedback regulation is common in numerous signaling pathways to keep these pathways in check and maintain homeostasis. In Hpo signaling, dMyc, a target of Yki, has been found to negatively regulate Yki both transcriptionally and post-translationally (Neto-Silva *et al.* 2010). Since cell proliferation rates in different regions of the wing disc are largely uniform toward the end of wing disc development (Schwank *et al.* 2011; Wartlick *et al.* 2011), we hypothesized that differentially expressed CycE may inhibit Yki targets to constrain its proproliferative potentials. To investigate how Yki and CycE cooperate to regulate tissue growth, we examined the effect of ectopic CycE on Yki activity reporters *Diap1-GFP* and *CycE-lacZ*. Interestingly, we found that CycE overexpression lowers transcriptional activities of *Diap1* and *CycE* (Figure 7, A and C, quantified in Figure 7, E and E’). The wild-type control or changing other proproliferative factors, such as Cyclin D (CycD), could not downregulate Yki target genes (Figure 7, B and D and Figure S7 in File S1). Taken together, our results reveal a negative feedback loop of CycE to itself and other Yki target genes to maintain balanced growth when Yki is activated.

**Figure 8:**
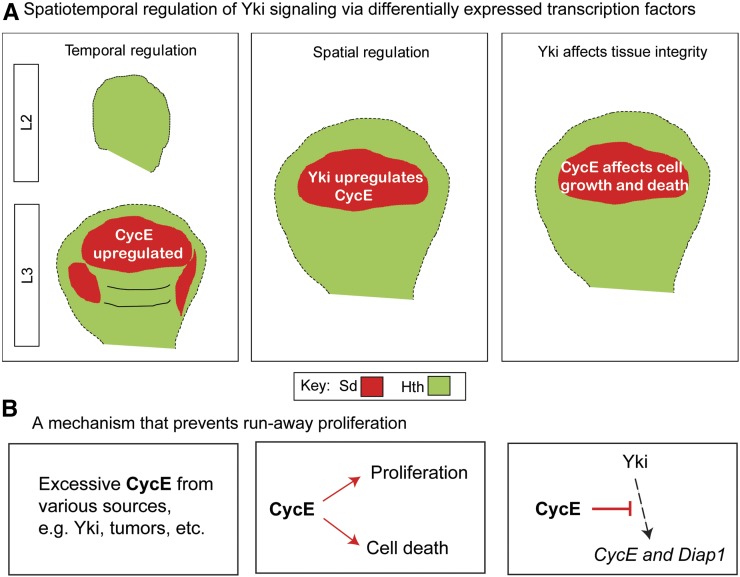
Spatiotemporal coordination between CycE and Yki maintains homeostasis. (A) During development, temporally controlled Sd determines upregulation of CycE after the L2 stage. In mature wing discs, spatially expressed Sd complexes with Yki to upregulate CycE in the pouch region. Spatiotemporally expressed Sd affects Yki regulation and further influences tissue integrity. (B) Excessive CycE inhibits Yki targets Diap1 and CycE to prevent run-away proliferation. CycE, Cyclin E; Sd, Scalloped; Yki, Yorkie.

The above data suggest a model in which Yki spatiotemporally regulates CycE through Sd (Figure 8A). This model predicts that Yki may differentially affect induced endoreplication in other tissues, depending on Sd availability. To corroborate this model, we quantified the NEIs of various genetic backgrounds in the eye imaginal disc. Due to the diverse cell fate of the eye disc cells, we only monitored undifferentiated proliferating cells that are anterior to the morphogenetic furrow (Figure S8A in File S1). Consistent with the model, addition of either CycE or Sd significantly reduced the NEIs of *Fzr*- and *Fzr/yki^M123^*-misexpressing cells (Figure S8, B–I in File S1). We further demonstrate a negative feedback loop involving CycE, Sd, and Yki. This may reveal a homeostasis mechanism that prevents runaway proliferation (Figure 8B).

## Discussion

In this study, we used the wing disc as a model system to characterize how differential regulation of proliferation by Yki is achieved. We have unraveled a means by which Yki, a key growth regulator, spatiotemporally regulates cell proliferation during organogenesis, and this differential regulation is dependent upon availability of its transcription factor partner Sd. As such, growth regulation by Yki discriminates cells on the basis of the location in the tissue and developmental stage. In fact, spatiotemporal control via transcription factors is found in a variety of biological processes, including the meiotic gene expression program, antioxidant and anti-inflammatory networks, and epithelial–mesenchymal transition-induced carcinoma metastasis (Tsai *et al.* 2012; Dinkova-Kostova *et al.* 2015; Alves-Rodrigues *et al.* 2016). We show that CycE is differentially upregulated by growth regulator Yki through spatiotemporally expressed transcription factor Sd. Interestingly, Sd has also been shown to be a default repressor, while Yki relieves this repression to promote growth (Koontz *et al.* 2013). Knockdown of Sd in Fzr-misexpressing cells also resulted in a decrease of the NEI (= 4.78), probably because repression on CycE expression was removed. However, our studies support the notion that Sd is required for Yki to upregulate CycE (Figure 4, B–B’’). Besides serving as transcription factors of Yki, both Sd and Hth have tissue-specific developmental roles in different tissues. During *Drosophila* wing development, Sd and Vg work together to control expression of wing-specific genes, such as *Sal* and *SRF*, and to promote wing formation (Halder *et al.* 1998; Simmonds *et al.* 1998). The interaction between Sd and Vg, a target of Notch signaling, thus provides a mechanism for Notch to inhibit Yki targets (Djiane *et al.* 2014). The development of *Drosophila* eyes requires Hth to complex with *eyeless* (*ey*) and *teashirt* (*tsh*), and this complex promotes cell proliferation (Bessa *et al.* 2002). Meanwhile, repression of Hth by the Dpp and Wingless (Wg) pathways in the wing pouch is a prerequisite for wing development (Wu and Cohen 1999; Casares and Mann 2000). Therefore, while Yki promotes tissue growth in a tissue-nonspecific manner, these distinct developmental roles of Sd and Hth dictate the availability of these two genes across different regions during different developmental stages (Wu *et al.* 2008; Zhang *et al.* 2008; Peng *et al.* 2009).

Differential control of CycE is critical to achieve tissue integrity (Bertoli *et al.* 2013). In multicellular organisms, tissues experience various stresses and damage from internal and external sources. To maintain integrity, two mechanisms have been identified during tissue repair: compensatory cell proliferation in proliferating tissues and compensatory cellular hypotrophy in postmitotic tissues (Huh *et al.* 2004, Ryoo *et al.* 2004; Tamori and Deng 2013, 2014). In response to tissue damage in proliferating tissues, such as *Drosophila* imaginal discs, Yki is activated by apoptosis through the Jun kinase pathway and therefore promotes cell proliferation (Sun and Irvine 2011). This elevated proliferation very likely requires upregulation of CycE. Indeed, a recent study showed that Sd and Yki drive CycE expression to induce cell cycle reentry in the eye disc in response to tissue damage (Meserve and Duronio 2015). In postmitotic tissues, such as *Drosophila* abdominal epithelia, injury leads to cell fusion and polyploidization controlled by Yki (Losick *et al.* 2013). This polyploidization requires a fluctuating level of CycE and thus Yki is unlikely to upregulate CycE uniformly in postmitotic tissues (Zielke *et al.* 2008). Comparing these two scenarios, we argue that CycE has to be differentially regulated by Yki to ensure that these two different yet connected biological processes take place. More work remains to be done to determine whether the Sd expression level plays an important role in these tissues.

While proper regulation of CycE is obviously crucial to the cell cycle, a growing number of studies have focused on its roles in cancer. Dysregulation of CycE induces chromosomal instability and has been found to be highly correlated with cancers in the lung, liver, intestine, brain/spine, bone, and breast (Bortner and Rosenberg 1997; Donnellan and Chetty 1999; Malumbres and Barbacid 2001). Our findings show that Sd is critical for Yki to elevate CycE, suggesting Sd to be an effective target in controlling CycE. In fact, the TEAD/TEF family proteins, the homolog of Sd in mammals, have recently been reported to have high expressions in various cancers and are implicated in their oncogenic roles in promoting tumorigenesis (Zhou *et al.* 2016). A dominant-negative TEAD molecule can suppress tumorigenesis from YAP overexpression (Liu-Chittenden *et al.* 2012). Therefore, differentially regulated CycE is critically important to a broad range of biological processes during development.

Interestingly, our results unveil a new regulatory mechanism in maintaining the levels of Yki target genes in which excessive CycE negatively feeds back Hpo signaling, resulting in a reduction in CycE and Diap1 expression. Negative feedback loops are not uncommon in Hpo signaling. Yki transcriptionally upregulates *ex*, *kibra*, and *crb*, upstream apical proteins in Hpo signaling, and these proteins then promotes nuclear exclusion of Yki to dampen its activities (Hamaratoglu *et al.* 2006; Genevet *et al.* 2009; Ling *et al.* 2010; Yu *et al.* 2010). Yki was recently found to induce transcription of Warts, a core component in Hippo signaling and a Yki upstream negative regulator, and this negative feedback loop is also conserved in mammals (Park *et al.* 2016). Our results show that Yki target CycE, a cell cycle regulator, can also negatively regulate Hpo signaling. It seems that this feedback regulation works downstream of Yki, because our preliminary genetics data show that overexpression of Yki fails to compensate for the downregulation of Diap1 by excessive CycE. Therefore, it would be very interesting to investigate how CycE affects Yki targets.

The differential regulation of CycE by Yki and negative feedback in the pouch seem to suggest that the maintenance and regulation of the pouch region of the wing imaginal disc is more complicated than previously thought. During pupal morphogenesis, the pouch epithelial cells undergo drastic morphological changes and basally extrude to form the wing blade (Waddington 1941). Therefore, the intrinsic complexity of the pouch ensures a finer control and correction against errors. A longstanding question in the tumor field is where and how malignant tumors start. Recent studies on tumorigenesis and cell competition show that the pouch region is a hot spot of cell competition, a process during which unfit cells are eliminated, and a cold spot of tumorigenesis, due to its unique cellular architecture and JAK/STAT signaling (Tamori and Deng 2011; Tamori *et al.* 2016). However, deregulation of Hpo signaling induces hyperplastic overgrowth and benign tumors (Justice *et al.* 1995). Our research reveals a mechanism by which the pouch region refrains from developing this type of tumor. As Hpo signaling is also linked to other signaling pathways, *e.g.*, JNK, Notch, EGFR, and cell competition (Yu *et al.* 2008; Chen *et al.* 2012; Reddy and Irvine 2013; Sun and Irvine 2013), it will be interesting to investigate whether CycE cross-talks with other pathways and is involved in cell competition.

## Supplementary Material

Supplemental material is available online at www.g3journal.org/lookup/suppl/doi:10.1534/g3.117.039065/-/DC1
