# Supplementary material for: Differential Regulation of Cyclin E by Yorkie-Scalloped Signaling in Organ Development
Source: G3 (Bethesda). 2017 Jan 30;7(3):1049–60. doi: 10.1534/g3.117.039065 (PMC5345706; doi:10.1534/g3.117.039065)
Supplement: Supplementary file 1 [file 1049FileS1.pdf]

Figure S1

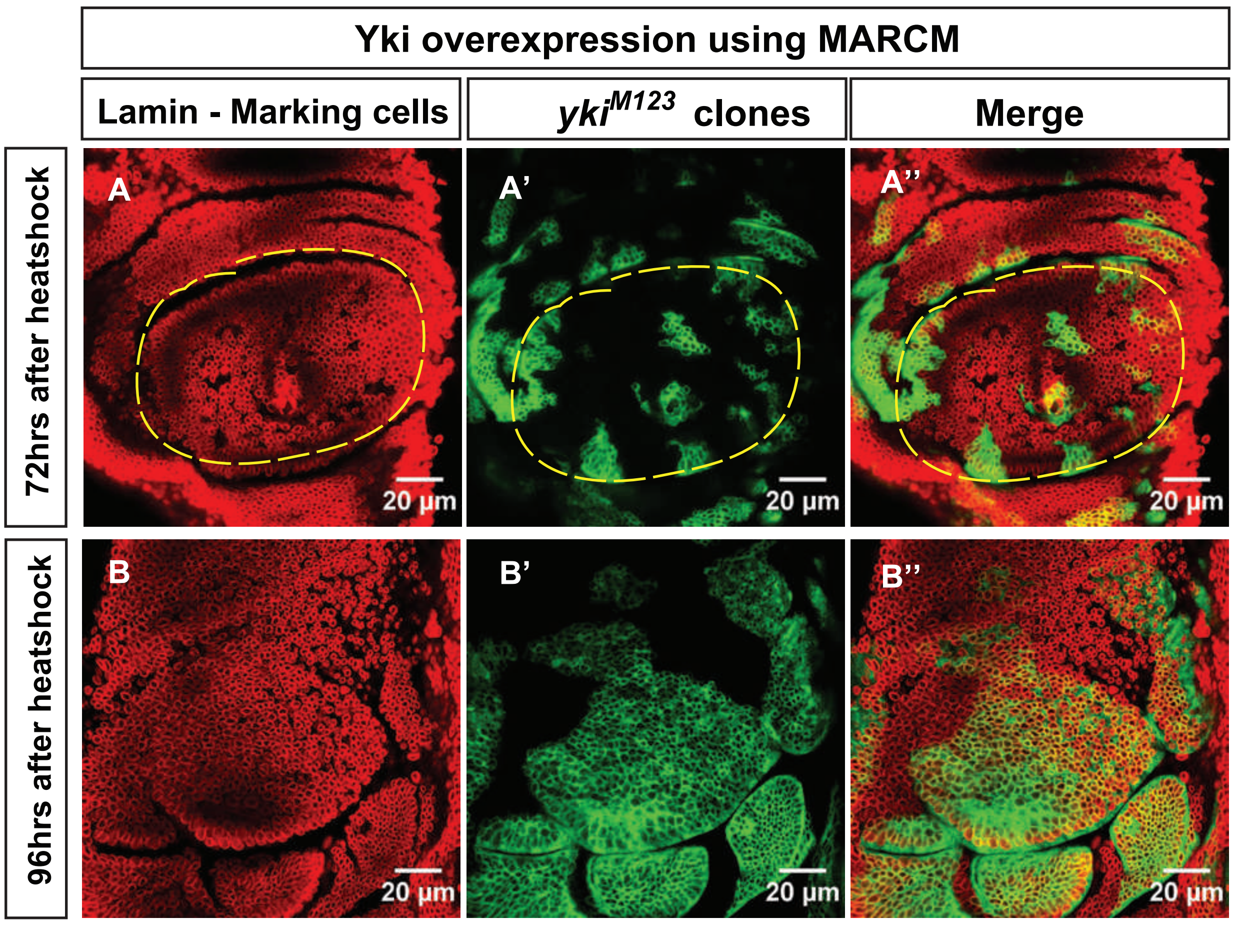

Figure S2

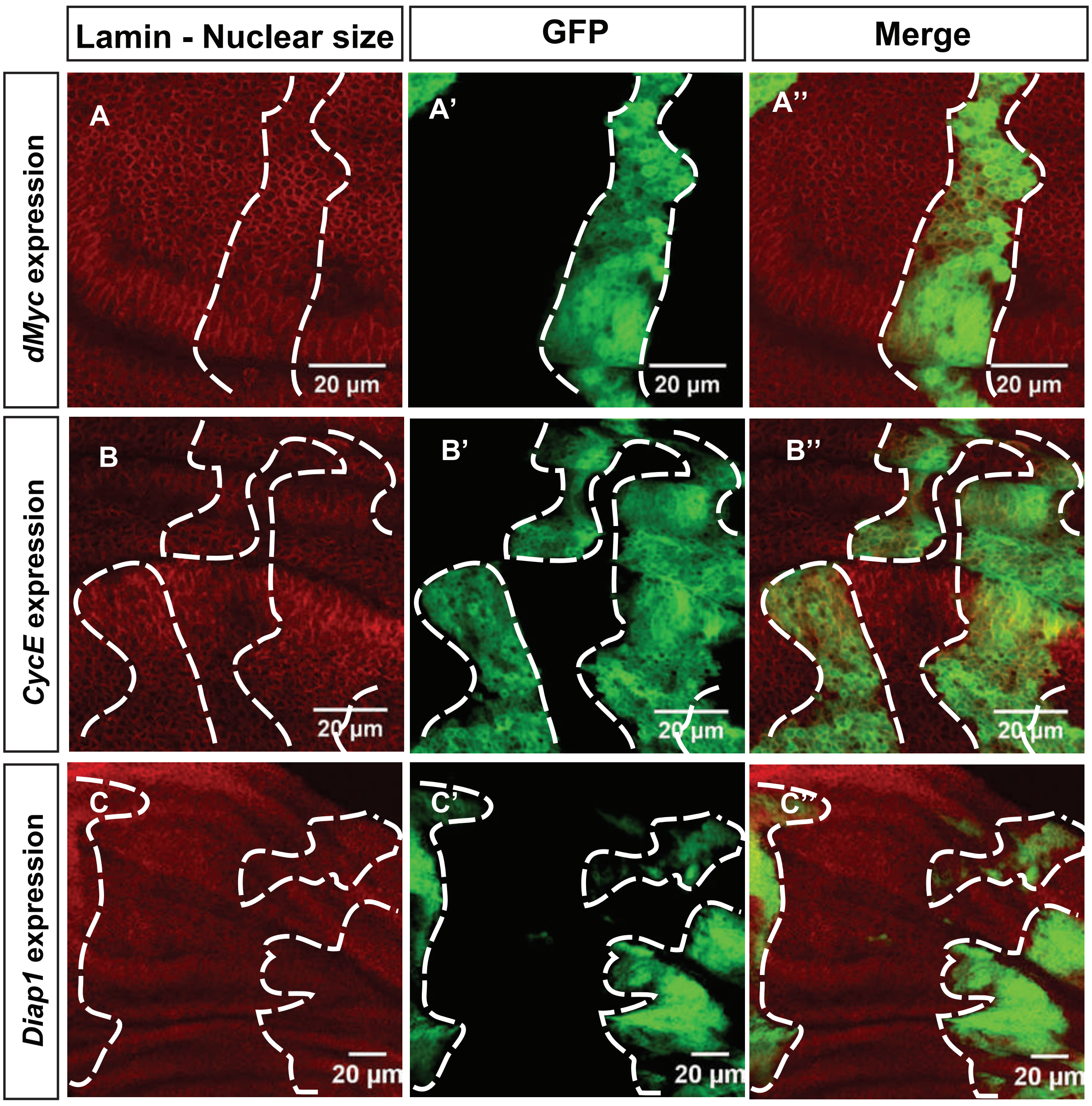

Table S1

Binding events of Sd on *CycE* sequence and 16.4 kb 5' *CycE* regulatory sequence

Transcription Factors	Chromosome	Peak Start	Peak End	Putative Target Genes
**Sd**	2L	15742935	15745542	FBgn0010382
	2L	15745543	15746281	FBgn0010382
	2L	15746282	15746468	FBgn0010382
	2L	15746543	15747159	FBgn0010382
	2L	15747160	15747546	FBgn0010382
	2L	15747909	15748183	FBgn0010382
	2L	15762150	15762826	FBgn0001987
**Hth**	2L	15743536	15744084	FBgn0010382
	2L	15746653	15747159	FBgn0010382
	2L	15747160	15747707	FBgn0010382
	2L	15747947	15748221	FBgn0010382

Note:
CycE sequence and 16.4 kb of the 5' *CycE* regulatory sequence: 15727581 - 15764550
FBgn0010382: *Cyclin E*

Figure S3

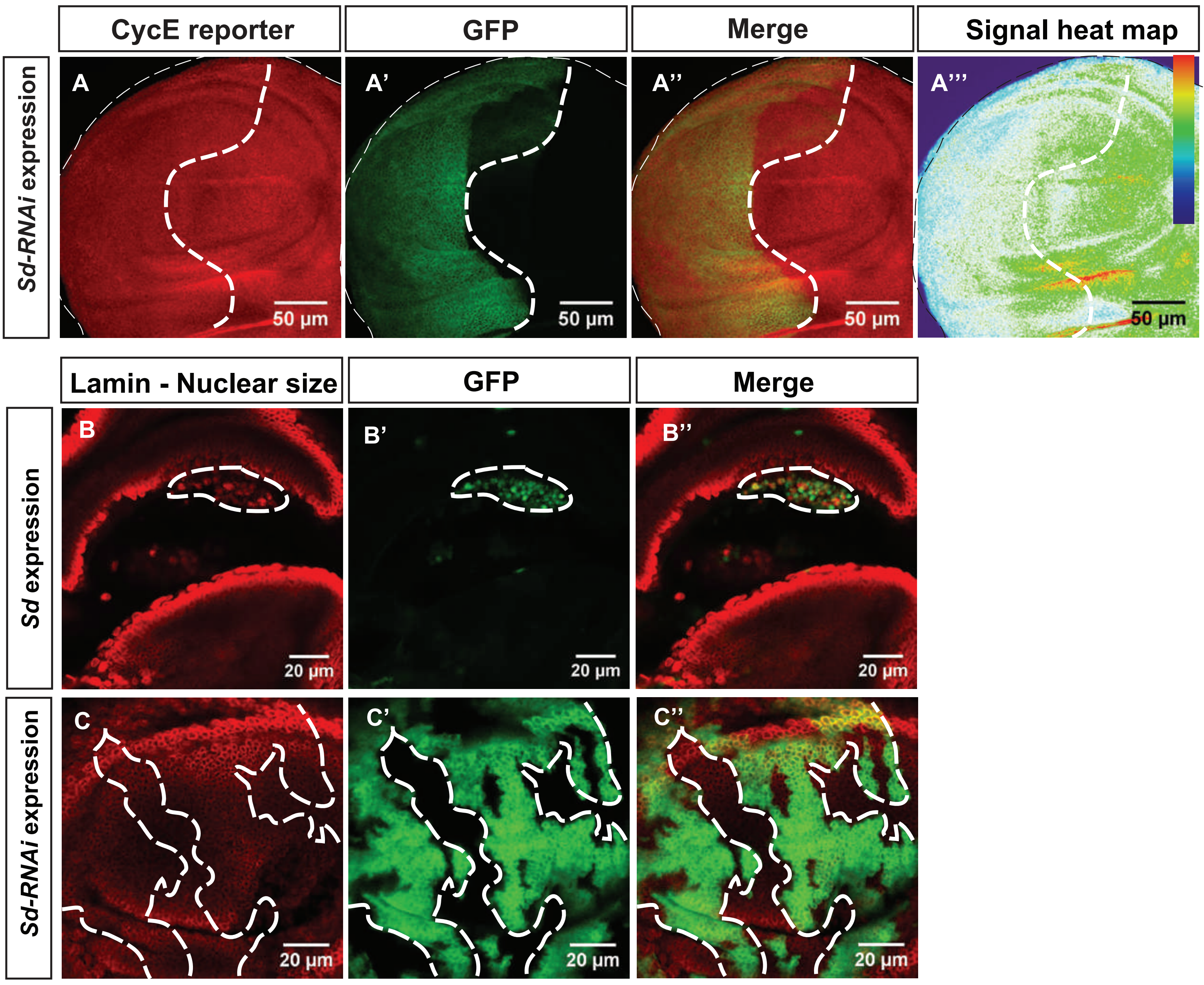

Figure S4

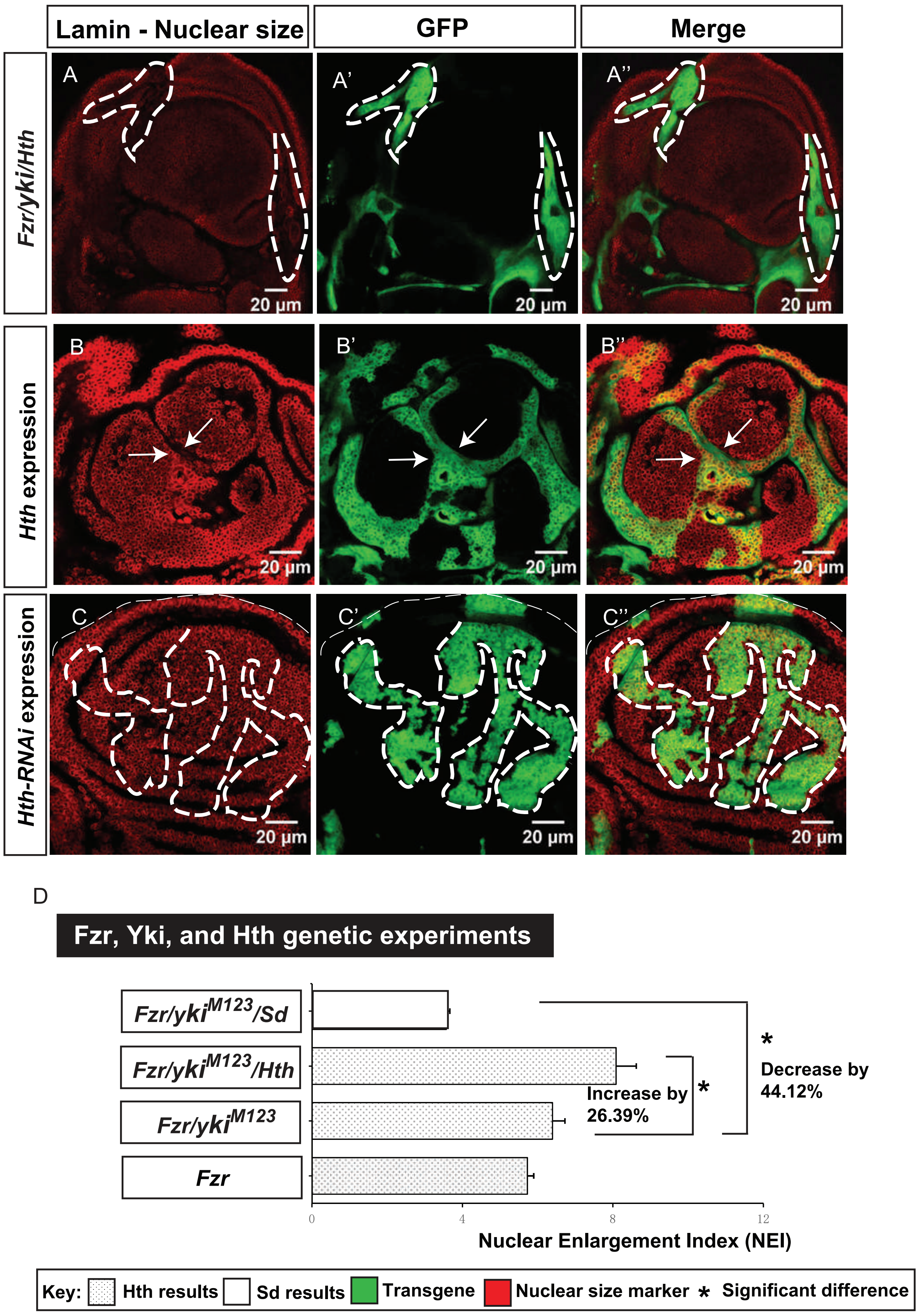

Figure S5

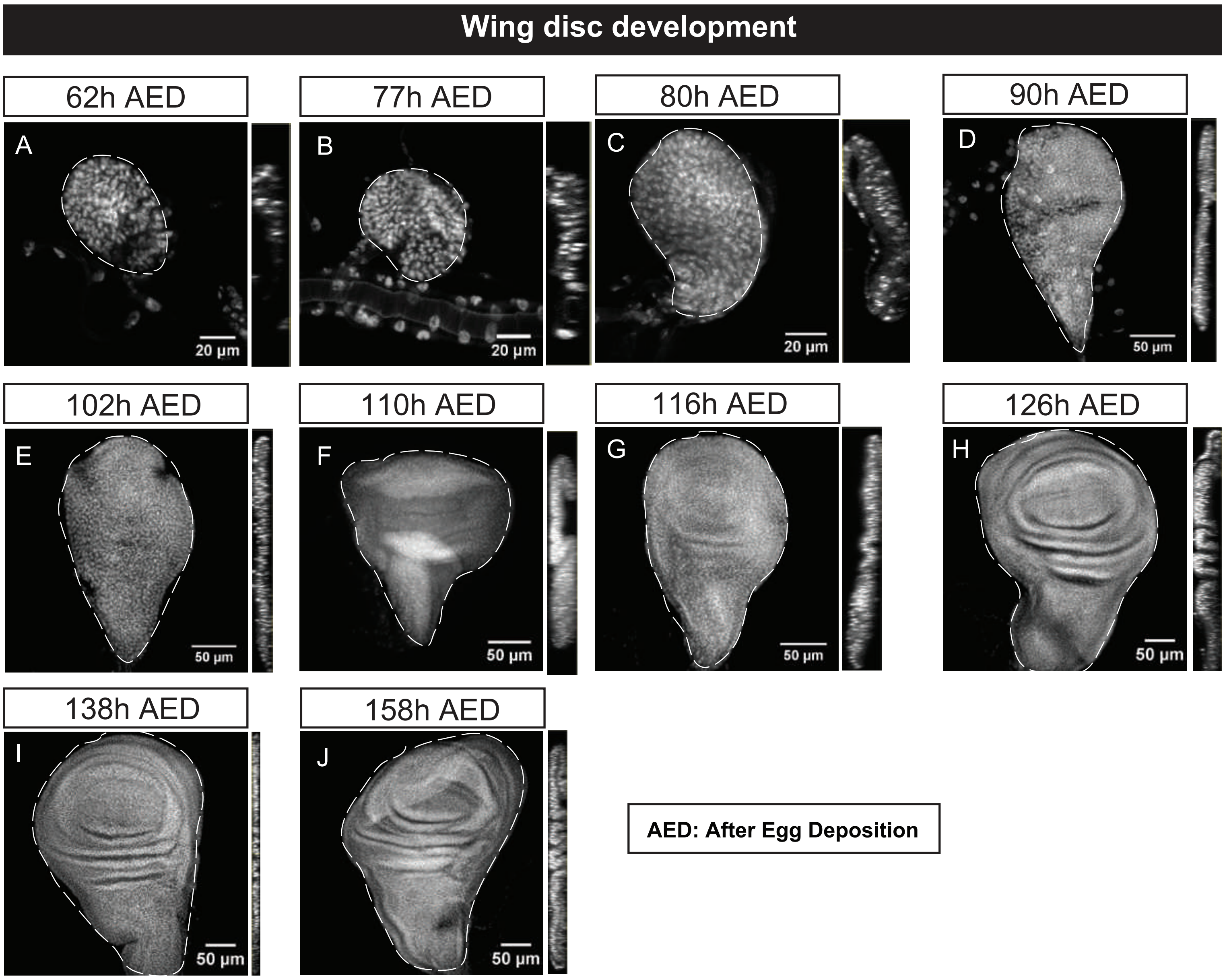

Figure S6

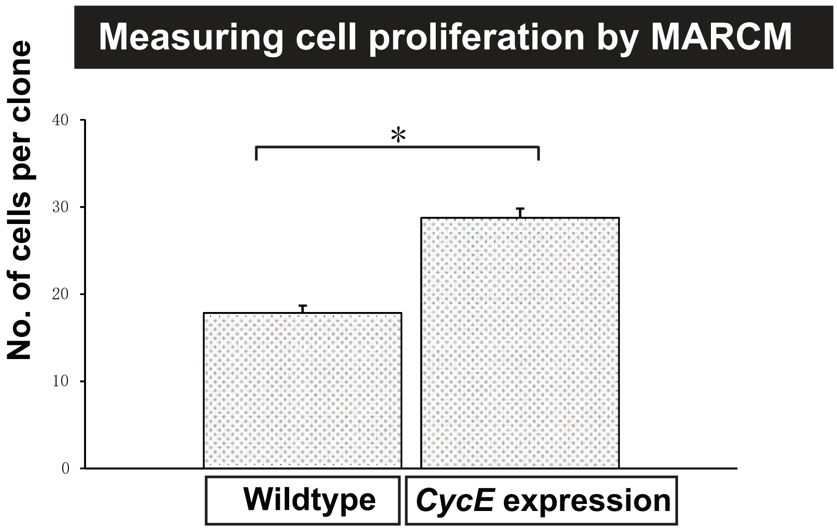

Figure S7

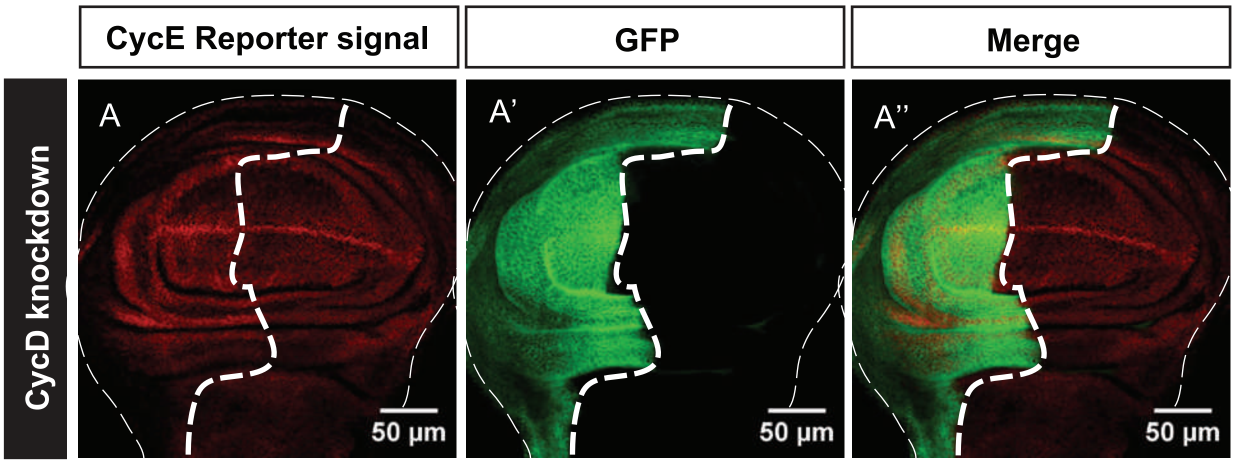

Figure S8

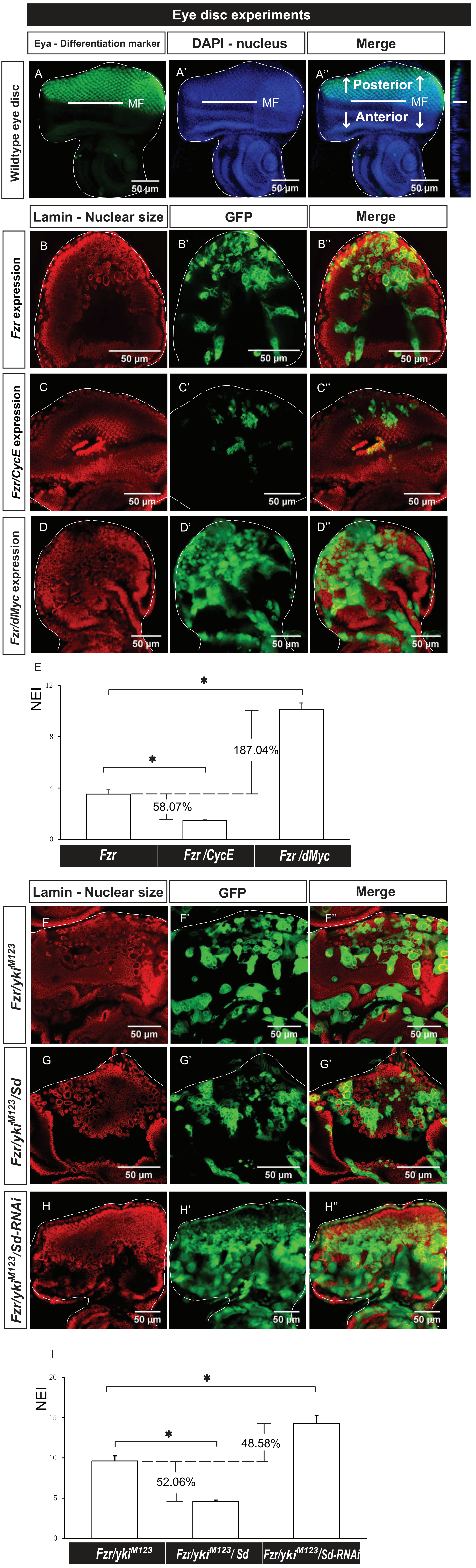
Eye disc experiments
Eya - Differentiation marker
DAPI - nucleus
Merge
Wildtype eye disc
A
A'
A''
MF
Posterior
Anterior
50 µm
Lamin - Nuclear size
GFP
Merge
Fzr expression
Fzr/CycE expression
Fzr/dMyc expression
B
B'
B''
C
C'
C''
D
D'
D''
E
NEI
12
8
4
0
*
187.04%
58.07%
Fzr
Fzr /CycE
Fzr /dMyc
Lamin - Nuclear size
GFP
Merge
Fzr/yki^M123
Fzr/yki^M123/Sd
Fzr/yki^M123/Sd-RNAi
F
F'
F''
G
G'
H
H'
H''
I
NEI
20
15
10
5
0
*
48.58%
52.06%
Fzr/yki^M123
Fzr/yki^M123/ Sd
Fzr/yki^M123/Sd-RNAi
