# Supplementary material for: Differential Regulation of Cyclin E by Yorkie-Scalloped Signaling in Organ Development
Source: G3 (Bethesda). 2017 Jan 30;7(3):1049–60. doi: 10.1534/g3.117.039065 (PMC5345706; doi:10.1534/g3.117.039065)
Supplement: Supplementary file 2 [file 1049FileS2.docx]

**Supporting information**

**Figure S1. Activation of Yki promotes proliferation.**

(A-B’’) The proliferation effect of Yki overexpression was examined using the MARCM technique 72 hours (A-A’’) and 96 hours (B-B’’) after heat-shock. Yki overexpression induces more cells and ectopic epithelial folds (B-B’’). Yellow dotted lines outline the wing pouch region. The scale bar is 20 cm.

**Figure S2. Overexpression of dMyc, CycE, or DIap1 does not affect the nuclear size.**

(A-C’’) Overexpression of dMyc, Diap1, or CycE does not affect the nuclear size. White dotted lines outline transgene expressing cells. The scale bar is 20 cm.

**Table S1. Available RNA-seq data reveal Sd DNA binding sites on the *CycE* locus.**

Published RNA-seq data (Slattery *et al*. 2013) indicate seven putative Sd DNA binding sites within the sequence of *CycE* or *CycE* regulatory region (16.4 kb).

**Figure S3. Effects of Sd in the wing disc.**

(A-A’’’) Sd overexpression shows mild downregulation of the CycE reporter *CycE-lacZ* in the wing disc. Thin white dotted lines outline the wing disc, and thick white dotted lines separate transgene expressing cells and wildtype cells. The scale bar is 50 cm

(B-C’’) Overexpression or knockdown of Sd does not affect the nuclear size. White dotted lines outline transgene expressing cells. The scale bar is 20 cm.

**Figure S4. Hth does not inhibit Fzr-induced endoreplication, likely due to its inability to upregulate CycE.**

(A-A’’) Overexpression of *Fzr/yki^M123^/Hth* was examined in the wing disc. White dotted lines outline transgene expressing cells. The scale bar is 20 cm

(B-C’’) Overexpression and knockdown of Hth were examined. Overexpression of Hth induces ectopic folds in the wing pouch (indicated by arrows), while Hth-RNAi does not change the morphology of hinge cells (circled by white dotted lines).

(D) Quantifications of NEIs of Fzr, Yki, and Hth genetic experiments are shown. Overexpression of Hth fails to reduce NEI of *Fzr/yki^M123^*, suggesting its inability to upregulate CycE. In contrast, overexpression of Sd significantly decreases NEI of Fzr/yki. (n(*Fzr*) = 62; n(*Fzr/yki^M123^*) = 31; n(*Fzr/yki^M123^/Hth*) = 10; n(*Fzr/yki^M123^/Sd*) = 96, error bars are standard error values).

**Figure S5. A time course study to trace wing disc development.**

(A-J) Pictures of developing wing discs at different time points (hours after egg deposition) were taken. The transverse view was also captured and is placed right next to the front view. White dotted lines outline entire discs. The scale bar is 20 cm (62h, 77h, and 80h AED) and 50 cm (the rest).

**Figure S6. Activation of CycE promotes proliferation**

Quantifications of the clonal cell number of wildtype and CycE ectopic expression using the MARCM technique (Lee and Luo 2001), demonstrating that the average number of cells per clone is significantly larger when CycE is overexpressed (n(wildtype) = 45; n(*CycE*) = 36, error bars are standard error values)

**Figure S7. CycD, a growth regulator and cell cycle gene, does not inhibit the CycE reporter**

(A-A’’) Overexpression of CycD does not affect CycE-lacZ in the wing disc. Thin white dotted lines outline the wing disc, and thick white dotted lines outline the boundary between CycD expressing cells and wildtype cells. The scale bar is 50 cm.

**Figure S8. Sd and CycE are capable of suppressing Fzr-induced endoreplication in the eye disc.**

(A-A’’) A wildtype eye disc was examined, with Eya marking differentiated cells. White lines indicate the morphegenetic furrow (MF) and arrows mark posterior and anterior compartment of the eye disc

(B-D’’) Overexpression of *Fzr*, *Fzr/CycE* and *Fzr/dMyc* were expressed in the eye disc.

(E) Quantifications of NEIs of *Fzr*, *Fzr/CycE*, and *Fzr/dMyc* are shown. CycE significantly suppresses Fzr-induced endoreplication by 58.07%, while dMyc significantly increases endoreplication by 187.04% (n(*Fzr*) = 26; n(*Fzr/CycE*) = 26; n(*Fzr/dMyc*) = 39, error bars are standard error values).

(F-H’’) Overexpression of *Fzr/yki^M123^*, *Fzr/yki^M123^/Sd*, and *Fzr/yki^M123^/Sd-RNAi* were examined in the eye disc.

(I) Quantifications of NEIs of the Fzr, Yki and Hth experiments are shown. Sd significantly reduces NEI of *Fzr/yki^M123^* by 52.06%, while *Sd-RNAi* significantly increases its NEI by 48.58% (n(*Fzr/yki^M123^*) = 27; n(*Fzr/yki^M123^/Sd*) = 38; n(*Fzr/yki^M123^/Sd-RNAi*) = 20, error bars are standard error values).

White dotted lines outline the eye discs. The scale bar is 50 cm.

**Detailed genotypes for each experiment in the figures**

Figure 1

**C.** *hsFlp; act>CD2>Gal4/UAS-Fzr; UAS-Yki ^M123^/+*

**D.** *hsFlp; act>CD2>Gal4/UAS-Fzr; +/+*

Figure 2

**A*.*** *CycE-lacZ/+; Hh-Gal4, UAS-GFP/UAS-yki ^M123^*

**B.** *CycE-lacZ/+; Hh-Gal4, UAS-GFP/+*

**C.** *en-Gal4, UAS-RFP; Diap1-GFP/UAS-yki ^M123^*

**D.** *en-Gal4, UAS-RFP; Diap1-GFP/+*

**E.** *ex-lacZ/+; Hh-Gal4, UAS-GFP/UAS-yki ^M123^*

**F.** *ex-lacZ/+; Hh-Gal4, UAS-GFP/+*

Figure 3

**A.** *hsFlp; act>CD2>Gal4, UAS-GFP/+; UAS-Fzr/UAS-dMyc*

**B.** *hsFlp; act>CD2>Gal4, UAS-GFP/+; UAS-Fzr/UAS-Diap1*

**C.** *hsFlp; act>CD2>Gal4, UAS-GFP/+; UAS-Fzr/UAS-CycE*

**E.** *hsFlp; act>CD2>Gal4, UAS-GFP/UAS-CycE; UAS-Fzr/UAS-yki ^M123^*

Figure 4

**A.** *Hth:YFP*

**B.** *CycE-lacZ/UAS-Sd-RNAi; Hh-Gal4/, UAS-GFP/UAS-yki ^M123^*

**C.** *hsFlp; act>CD2>Gal4, UAS-GFP/UAS-Sd; UAS-Fzr/+*

**D.** *hsFlp; act>CD2>Gal4, UAS-GFP/UAS-Sd-RNAi; UAS-Fzr/+*

**E.** *hsFlp; act>CD2>Gal4, UAS-GFP/UAS-Sd; UAS-Fzr/UAS-yki ^M123^*

**F.** *hsFlp; act>CD2>Gal4/, UAS-GFP/UAS-Sd-RNAi; UAS-Fzr/UAS-yki ^M123^*

Figure 5

**C-F.** *Hth:YFP*

**H.** *hsFlp; act>CD2>Gal4, UAS-GFP /+; UAS-Fzr/UAS-yki ^M123^*

**I.** *hsFlp; act>CD2>Gal4, UAS-GFP /+; UAS-Fzr/UAS-yki ^M123^/UAS-Sd*

Figure 6

**A-C.** *en-Gal4, UAS-GFP/+; UAS-CycE/tubP-Gal80^ts^*

Figure 7

**A**. *hsFlp; act>CD2>Gal4, UAS-RFP; Diap1-GFP/UAS-CycE*

**B**. *en-Gal4, UAS-RFP; Diap1-GFP/+*

**C**. *hsFlp; CycE-lacZ/+; act>CD2>Gal4, UAS-RFP/UAS-CycE*

**D**. *CycE-lacZ/+; Hh-Gal4, UAS-GFP/+*

Figure S1

**A-B.** *UAS-CD8-GFP, hsFlp; FRT42D ubi:Gal80/FRT42D +; tublin-Gal4/UAS-yki* ^M123^

Figure S2

**A.** *hsFlp; act>CD2>Gal4, UAS-GFP/+; UAS-dMyc/+*

**B.** *hsFlp; act>CD2>Gal4, UAS-GFP/+; UAS-CycE/+*

**C.** *hsFlp; act>CD2>Gal4, UAS-GFP/+; UAS-Diap1/+*

Figure S3

**A.** *CycE-lacZ/+; Hh-Gal4, UAS-GFP/UAS-Sd-RNAi*

**B.** *hsFlp; act>CD2>Gal4, UAS-GFP/UAS-Sd*

**C.** *hsFlp; act>CD2>Gal4, UAS-GFP/UAS-Sd-RNAi*

Figure S4

**A.** *hsFlp; act>CD2>Gal4, UAS-GFP/UAS-yki; UAS-Fzr/UAS-Hth*

**B.** *hsFlp; act>CD2>Gal4, UAS-GFP/+; UAS-Hth/+*

**C.** *hsFlp; act>CD2>Gal4, UAS-GFP/+; UAS-Hth-RNAi/+*

Figure S6

*UAS-CD8-GFP, hsFlp; FRT42D ubi:Gal80/FRT42D +; tublin-Gal4/UAS-CycE*

Figure S7

**A**. *hsFlp; act>CD2>Gal4, UAS-GFP/UAS-CycD*

Figure S8

**B.** *hsFlp; act>CD2>Gal4, UAS-GFP/+; UAS-Fzr/+*

**C.** *hsFlp; act>CD2>Gal4, UAS-GFP/+; UAS-Fzr/UAS-CycE*

**D.** *hsFlp; act>CD2>Gal4, UAS-GFP/+; UAS-Fzr/UAS-dMyc*

**F.** *hsFlp; act>CD2>Gal4, UAS-GFP/+; UAS-Fzr/UAS-yki ^M123^*

**G.** *hsFlp; act>CD2>Gal4, UAS-GFP/UAS-Sd; UAS-Fzr/UAS-yki ^M123^*

**H.** *hsFlp; act>CD2>Gal4, UAS-GFP/UAS-Sd-KD; UAS-Fzr/UAS-yki ^M123^*
